# Association of Carotid Atherosclerosis With Lipid Components in Asymptomatic Low-Income Chinese: A Population-Based Cross-Sectional Study

**DOI:** 10.3389/fneur.2020.00276

**Published:** 2020-04-24

**Authors:** Jing Pan, Jie Liu, Hong Wang, Weilan Li, Xin Du, Qiuxing Lin, Xinxin Zhang, Dongwang Qi, Jun Tu, Xianjia Ning, Qing Yang, Jinghua Wang

**Affiliations:** ^1^Department of Neurology, Tianjin Medical University General Hospital, Tianjin, China; ^2^Laboratory of Epidemiology, Tianjin Neurological Institute, Tianjin, China; ^3^Key Laboratory of Post-Neuroinjury Neuro-repair and Regeneration in Central Nervous System, Tianjin Neurological Institute, Tianjin, China; ^4^Tianjin Academy of Traditional Chinese Medicine Affiliated Hospital, Tianjin, China; ^5^Department of Cardiology, Tianjin Medical University General Hospital, Tianjin, China; ^6^Department of Endocrinology and Metabolism, Tianjin Medical University General Hospital, Tianjin, China

**Keywords:** lipids, carotid intima-media thickness, carotid plaque, risk factors, ultrasonography, atherosclerosis

## Abstract

Intima-media thickness is a non-invasive arterial marker of early-stage atherosclerosis. Identifying carotid plaque is a superior surrogate endpoint for assessing atherosclerotic lesions. The aim of this study was to investigate the association of carotid intima-media thickness (CIMT) and carotid plaque with lipids among asymptomatic low-income rural residents in China. A total of 3,789 people aged ≥45 years without a history of stroke or cardiovascular disease were recruited to this study. B-mode ultrasonography was performed to measure CIMT and identify carotid plaque for early identification of atherosclerosis. Multivariate analysis was used to assess the association of blood lipid levels with atherosclerosis. The mean CIMT across our cohort was 567 μm. A linear regression analysis showed that low-density lipoprotein cholesterol (LDL-C) and total cholesterol (TC) were risk factors for early-stage atherosclerosis; however, high-density lipoprotein cholesterol and triglycerides protected against early-stage atherosclerosis after adjusting for potential risk factors (*P* < 0.001). Carotid plaque risk increased by 24 and 62% for each 1-mmol/L increase in TC and LDL-C (*P* < 0.001). These findings suggest that it is vital to manage and control the dyslipidemia standard levels in China, especially among rural residents, in order to reduce the burden of cardiovascular diseases.

## Introduction

Stroke was the leading cause of death and the third most common cause of disability in China between 1990 and 2017 (1, 2). The most common type of stroke is thrombotic stroke, and atherosclerosis is the most common cause (3). Thus, early detection and management of individuals at high risk is critical to prevent or delay otherwise inevitable end-events.

Several studies have shown that intracranial or extracranial atherosclerosis is a valid predictor of the presence of a cerebrovascular disease (CVD) or stroke (4). The measurement of carotid intima media thickness (CIMT) using high-resolution ultrasound is useful for detecting early atherosclerotic changes (5, 6). Moreover, previous studies have demonstrated that increased CIMT was a marker for early atherosclerosis (7, 8) and that carotid plaque was a better parameter for assessing atherosclerotic lesions (9, 10). In addition, the hazard ratio of myocardial infarction or stroke was 1.09 for every 0.1-mm increase in CIMT (11). Other studies have investigated the determinants of atherosclerosis, such as age, sex, current smoking, alcohol consumption, and education levels (12–15). The association of atherosclerosis with blood lipids has also been studied, but the results were controversial (16–18).

Considering the health consequences of atherosclerosis, further study of the effects of lipids on CIMT and carotid plaque is critical. However, to the best of our knowledge, few studies have examined this relationship, especially among low-income individuals with low educational levels who live in rural areas of China. Thus, we aimed to perform a population-based study to determine the association between blood lipids and different stages of atherosclerosis, including carotid plaque and CIMT, among a low-income population in rural China.

## Methods

### Participants and Study Design

This was a cross-sectional, population-based survey conducted in the rural areas of Tianjin, China. The study was performed from April 2014 to January 2015 using participants from the Tianjin Brain Study, which has been described previously (19). In brief, the entire population comprised of 14,251 participants from 18 administrative villages in rural Tianjin, China. About 95% of the participants were low-income farmers, with a per capita disposable annual income of less than US$1,600 in 2014 (20). All residents aged 45 years and older without CVDs were recruited to this study, while those with a history of CVD were excluded. Those with other vascular events were also excluded, including acute coronary events, any form of coronary artery disease, and peripheral artery disease. We determine CVD and other vascular events based on the patient's medical history and imaging data.

All investigations were approved by the ethics committee of Tianjin Medical University General Hospital. The study was carried out in accordance with the approved guidelines, and informed consent was obtained from all participants.

### Information Collection and Risk Factor Definitions

All data in this study were obtained by trained epidemiological researchers through face-to-face interviews based on a pre-specified questionnaire.

Demographic information, including name, sex, date of birth, and educational level, were retrieved from previous records. All participants were separated into four age groups: 45–54, 55–64, 65–74, and ≥75 years. Educational level was classified into three groups according to the length of each individual's formal education: illiteracy (without education), 1–6 years, and >6 years.

Previous individual and family medical histories, which included hypertension, diabetes mellitus (DM), stroke, transient ischemic attack, and coronary heart disease, were obtained by patient self-reporting or review of previous records.

Lifestyle characteristics included cigarette smoking and alcohol consumption. Cigarette smoking was defined as smoking more than one cigarette per day for at least 1 year, and the participants were categorized as never smokers and smokers. Alcohol consumption was defined as drinking more than 500 g f alcohol per week for at least 1 year, and the participants were divided into a never alcohol consumption group and an alcohol consumption group.

### Physical Examination

Blood pressure [including systolic blood pressure (SBP) and diastolic blood pressure (DBP)], height, weight, and circumference were measured at the local village clinic during the baseline survey. The levels of fasting blood glucose (FBG), total cholesterol (TC), triglycerides (TG), high-density lipoprotein cholesterol (HDL-C), and low-density lipoprotein cholesterol (LDL-C) in the serum were assessed at the Ji County People's Hospital. Carotid ultrasonography and 12-lead echocardiography were also conducted by a professional. Body mass index (BMI) was calculated as weight (kg) divided by the square of height (m^2^).

The sitting position BP was measured after abstaining from cigarettes, alcohol, tea, coffee, or physical activity for at least 30 min. In an effort to minimize white coat hypertension, BP measurements were performed by the researchers in a quiet room, using the standard method described by the American Hypertension Association. Two readings were recorded and the average value was calculated.

### Ultrasonography Measurements

One trained technician blinded to the participants' information performed the ultrasound examinations. The subjects were examined, while they were in a supine position, using B-mode ultrasonography (Terason 3000, Burlington, MA, USA) with a 5–12 MHz linear array transducer. The bilateral extracranial carotid artery trees (including the common carotid artery, carotid sinus, and internal and external carotid arteries) were screened for plaque. Carotid plaque is defined as: (1) local CIMT exceeds 1.5 mm, (2) localized CIMT bulge protrudes into the lumen for more than 0.5 mm, and (3) local CIMT exceeds 50% in the peripheral medial lining; early-stage atherosclerosis was defined as CIMT ≥1 mm (21, 22). CIMT at the near and far walls of the common carotid artery was measured on the left and the right, and three values were obtained: maximum CIMT, minimum CIMT, and average CIMT per lateral. The average CIMT was calculated according to the means for the sum of the CIMTs both on the left side and on the right side. The images were obtained and digitally stored according to a standard protocol (23).

### Survey Procedure

Local village physicians visited all eligible residents at their home according to a predefined protocol 1 day before the examination. We performed a physical examination (including blood pressure, weight, height, and circumference measurement, carotid ultrasonography, and 12-lead echocardiography) and collected blood samples at local village clinics between April and July in 2014. All blood samples were sent within 2 h of collection to the central laboratory at Tianjin Ji County People's Hospital for measurement of FBG, TC, TG, HDL-C, and LDL-C levels using standardized enzymic methods.

### Definition of Risk Factors

Hypertension was defined as SBP ≥140 mmHg, DBP ≥90 mmHg, or taking medication for hypertension. DM was defined as FBG concentration ≥7.0 mmol/L or taking medication for diabetes. Central obesity was defined as a waist circumference of >102 cm in men and greater than 88 cm in women (24). Obesity was defined as a BMI ≥28.0 kg/m^2^, and overweight was defined as a BMI of 24.0–27.9 kg/m^2^ (25).

### Statistical Analyses

Continuous variables, including age, TC, TG, HDL-C, and LDL-C, are presented as means and standard deviations; differences between groups are performed by Student's t-test. The categorical variables are presented as frequencies and 95% confidence intervals (CIs); differences between groups are compared using chi-square tests. The risk factors for CIMT and CP were assessed individually for men and women using logistic regression analyses; the results of the univariate analysis were presented as unadjusted βs (95% CIs) or OR (95% CIs). The association of lipids with CIMT and CP was performed by multivariate analysis; the results were presented as adjusted βs (95% CIs) or OR (95% CIs) after adjustment for significant covariates in the univariate analysis. Of these covariates, education group, hypertension, DM, obesity, current smoking, and alcohol consumption were analyzed as categorical variables. Age, levels of FBP, TC, TG, HDL-C, and LDL-C were analyzed as continuous variables. *P* < 0.05 was considered as statistically significant for dichotomous variables and categorical variables with three or four groups. SPSS for Windows (version 19.0; SPSS Inc., Chicago, IL, USA) was used for all analyses.

## Results

### Demographic Characteristics for Subjects

**Figure 1:**
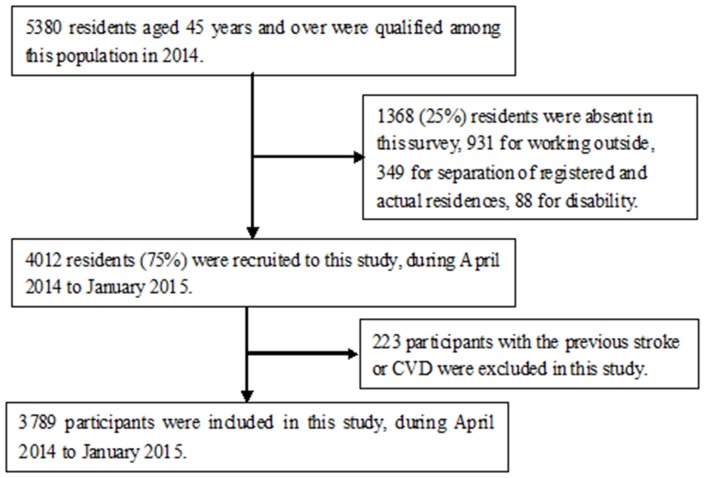
Flow chart of participants.

A total of 4,012 participants were interviewed from among 5,380 residents aged 45 years and older during the study period, excluding 1,368 residents who were absent during the survey due to working outside (*n* = 931), separation of registered and actual residences (*n* = 349), and disability (*n* = 88). Finally, 3,789 individuals [1,560 men (41.2%) and 2,229 women (58.8%); mean age, 59.92 years] were enrolled in our study after excluding 223 residents with a previous history of CVD or stroke (Figure 1). There were proportions of residents aged 45–54 years old among the absent group (57.2%) than that in this study (32.6%).

The education level of this population was very low; the average length of education was 5.48 years, and 17.4% of the participants had never received a formal education (8.8% of men and 23.4% of women). The prevalence of hypertension, DM, central obesity, current smoking, and alcohol consumption was 20.4, 14.1, 35.8, 34.0, and 14.3%, respectively. Moreover, the average SBP and DBP were high in this population, with mean values of 146.42 and 86.81 mmHg, respectively. The mean values of CIMT, TC, TG, HDL-C, and LDL-C were 567.13 μm, 4.87 mmol/L, 1.76 mmol/L, 1.46 mmol/L, and 2.70 mmol/L, respectively (Table 1).

**Table 1:** Characteristics of all participants in this study.

**Category**	**Total**	**Men**	**Women**
Total:	3,789 (100)	1,560 (41.2)	2,229 (58.8)
Age, means (SD), years	59.92 (9.70)	61.13 (9.90)	59.07 (9.47)
Age group, *n* (%)			
45–54 years	1,236 (32.6)	430 (27.6)	806 (36.2)
55–64 years	1,514 (40.0)	632 (40.5)	882 (39.6)
65–74 years	724 (19.1)	338 (21.7)	386 (17.3)
≥75 years	315 (8.3)	160 (10.3)	155 (7.0)
Education, means (SD), years	5.48 (6.54)	6.40 (3.22)	4.84 (3.61)
Education, *n* (%)			
0 years	659 (17.4)	137 (8.8)	522 (23.4)
1–6 years	1,694 (44.7)	699 (44.8)	995 (44.6)
> 6 years	1,436 (37.9)	724 (46.4)	712 (31.9)
Smoking status, *n* (%)			
Never smoking	2,842 (75.0)	743 (47.6)	2099 (94.2)
Ever smoking	173 (4.6)	146 (9.4)	27 (1.2)
Current smoking	774 (20.4)	671 (43.0)	103 (4.6)
Alcohol consumption, *n* (%)			
Never drinking	3,198 (84.4)	999 (64.0)	2199 (98.7)
Ever drinking	49 (1.3)	48 (3.1)	1 (0.0)
Current drinking	542 (14.3)	513 (32.9)	29 (1.3)
Hypertension, *n* (%)	2,583 (68.2)	1,111 (71.2)	1,472 (66.0)
Diabetes, *n* (%)	533 (14.1)	216 (14.1)	317 (14.5)
BMI, *n* (%)			
Normal	1,298 (34.3)	584 (37.4)	714 (32.0)
Overweight	1,603 (42.3)	653 (41.9)	950 (42.6)
Obesity	888 (23.4)	323 (20.7)	565 (25.3)
Central obesity, *n* (%)	3,040 (80.4)	1,116 (71.7)	1,924 (86.5)
SBP, means (SD), mmHg	146.42 (22.17)	147.76 (21.41)	145.49 (22.64)
DBP, means (SD), mmHg	86.81 (11.40)	88.50 (11.22)	85.62 (11.39)
BMI, means (SD), Kg/m^2^	25.57 (3.68)	25.20 (3.44)	25.82 (3.82)
FBG, means (SD), mmol/L	5.92 (1.57)	5.91 (1.42)	5.93 (1.67)
TC, means (SD), mmol/L	4.87 (1.09)	4.62 (1.00)	5.04 (1.11)
TG, means (SD), mmol/L	1.76 (1.24)	1.61 (1.24)	1.87 (1.22)
HDL-C, means (SD), mmol/L	1.46 (0.46)	1.39 (0.43)	1.50 (0.48)
LDL-C, means (SD), mmol/L	2.70 (1.25)	2.61 (1.20)	2.76 (1.28)
CIMT, means (SD), μm	567 (87.92)	583 (92.64)	556 (82.59)

*SD, standard deviation; SBP, systolic blood pressure; DBP, diastolic blood pressure; BMI, body mass index; FBG, fasting blood glucose; TC, total cholesterol; TG, triglycerides; HDL-C, high density lipoprotein cholesterol; LDL-C, low density lipoprotein cholesterol*.

### Association of Atherosclerosis With Conventional Risk Factors in the Univariate Analysis

The mean CIMT and carotid plaque prevalence were higher in men than in women and increased with increasing age but decreased with advancing educational levels (*P* < 0.001). There was a significantly higher mean CIMT and carotid plaque prevalence in current smokers, those with hypertension and diabetes, than their opposite (*P* < 0.001). Alcohol consumers associated with mean CIMT and BMI associated with carotid plaque prevalence (*P* < 0.001; Table 2).

**Table 2:** Relative factors of mean CIMT and carotid plaque prevalence in the univariate analysis.

**Risk factors**	**CIMT (μm**)		**Carotid plaque (%)**		
	**Means (SD)/ β (95%CI)**	***P***	**Carotid plaque**	**Non-carotid plaque**	***P***
Gender:		<0.001	1,574 (41.5)	2,215 (58.5)	<0.001
Men	583 (92.6)		782 (50.1)	778 (49.9)	
Women	556 (82.6)		792 (35.5)	1,437 (64.5)	
Age, years	2.52 (2.24, 2.80)	<0.001	63.38 (9.49)	57.45 (9.09)	<0.001
Age group:		<0.001			<0.001
45–54 years	536 (75.9)		281 (22.7)	955 (77.3)	
55–64 years	571 (85.3)		684 (45.2)	830 (54.8)	
65–74 years	593 (93.3)		390 (53.9)	334 (46.1)	
≥75 years	609 (91.9)		219 (69.5)	86 (30.5)	
Education:		<0.001			<0.001
0 years	584 (94.0)		318 (48.3)	341 (51.7)	
1–6 years	573 (87.9)		759 (44.8)	935 (55.2)	
> 6 years	552 (82.8)		497 (34.6)	939 (65.4)	
Smoking status:		<0.001			<0.001
Never smoking	556 (70.3)		1,121 (39.4)	1,721 (60.6)	
Ever smoking	575 (80.6)		75 (43.4)	98 (56.6)	
Current smoking	580 (94.7)		378 (48.8)	396 (51.2)	
Alcohol consumption:		<0.001			0.255
Never drinking	564 (85.4)		1,311 (41.0)	1,887 (59.0)	
Ever drinking	597 (89.3)		31 (63.3)	18 (36.7)	
Current drinking	585 (99.3)		232 (42.8)	310 (57.2)	
Hypertension:		<0.001			<0.001
Yes	579 (87.9)		1,213 (47.0)	1,370 (53.0)	
No	541 (79.3)		361 (29.9)	845 (70.1)	
Diabetes:		<0.001			<0.001
Yes	580 (85.6)		282 (52.9)	251 (47.1)	
No	565 (88.2)		1,263 (39.6)	1,929 (60.4)	
BMI:		0.318			0.015
Normal	565 (88.7)		577 (44.5)	721 (55.5)	
Overweight	567 (89.4)		646 (40.3)	957 (59.7)	
Obesity	571 (84.0)		351 (39.5)	537 (60.5)	
Central obesity		0.156			<0.001
Yes	565 (82.9)		510 (37.7)	843 (62.3)	
No	569 (90.6)		1,061 (43.7)	1,367 (56.3)	

Moreover, CIMT increased with the increasing level of TC and LDL-C, while it decreased with the increasing level of TG and HDL-C. Moreover, the levels of TC and LDL-C were greater among the participants with carotid plaque than those without carotid plaque (Table 3).

**Table 3:** Association of mean CIMT and carotid plaque with lipids in the univariate analysis.

**Lipids components**	**CIMT (μm)**		**Carotid plaque [means (SD)]**		
	**β** **(95%CI)**	***P***	**Carotid** **plaque**	**Non-carotid plaque**	***P***
TC	3.40 (0.80, 5.99)	0.010	4.99 (1.15)	4.78 (1.04)	<0.001
TG	−2.97 (−5.25, −0.69)	0.011	1.76 (1.13)	1.76 (1.31)	0.901
HDL-C	−6.51 (−12.65, −0.37)	0.037	1.45 (0.45)	1.46 (0.47)	0.582
LDL-C	6.57 (4.32, 8.82)	<0.001	3.07 (1.44)	2.43 (1.02)	<0.001

### Association of Atherosclerosis With Lipids in the Multivariate Analysis

Table 4 shows that the levels of TC, TG, HDL-C, and LDL-C associated independently with the mean CIMT after adjustment for other risk factors which are significant in the univariate analysis. The mean CIMT increased to 5.79 μm for each 1-mmol/L increase of TC (β, 5.79; 95% CI, 3.04–8.55; *P* < 0.001). Similarly, each 1-mmol/L increase of the LDL-C level resulted in a 5.07-μm increase of CIMT (β, 5.07; 95% CI, 2.93–7.21; *P* < 0.001). Oppositely, each 1-mmol/L increase of TG and HDL-C levels resulted in 4.84- and 12.20-μm decrease of CIMT (*P* < 0.001). Moreover, with each 1-mmol/L increase of TC and LDL-C, the risk of occurrence of carotid plaque increases by 24% (OR: 1.24, 95% CI: 1.16–1.32; *P* < 0.001) and by 62% (OR: 1.62, 95% CI: 1.51–1.73; *P* < 0.001), respectively.

**Table 4:** Association of mean CIMT and carotid plaque with lipids in the multivariate analysis.

**Lipids components**	**CIMT (μm)**		**Carotid plaque**		
	**β** **(95%CI)**	***P***	**References**	**OR (95%CI)**	***P***
TC	5.79 (3.04, 8.55)	<0.001	—	1.24 (1.15, 1.36)	<0.001
TG	−4.84 (−7.26, −2.42)	<0.001	—	—	—
HDL-C	−12.20 (−18.61, −5.79)	<0.001	—	—	—
LDL-C	5.07 (2.93, 7.21)	<0.001	—	1.62 (1.51, 1.73)	<0.001

*Model CIMT: including TC, TG, HDL-C, and LDL-C adjusted by statistical significance variates in the univariate analysis. Model carotid plaque: including TC and LDL-C adjusted by significant variates in the univariate analysis*.

## Discussion

This is the first population-based study to explore the association between lipids and different stages of atherosclerosis among a Chinese rural population with a high incidence of stroke. An early manifestation of atherosclerosis was CIMT thickening, while plaque formation was a late sign of atherosclerosis. In this low-income population, the levels of TC, TG, HDL-C, and LDL-C associated significantly with mean CIMT. High levels of TC and LDL-C and low levels of TG and HDL-C were the independent risk factors of early atherosclerosis. Nevertheless, an increase of the levels of TC and LDL-C associated with a higher risk of late-stage atherosclerosis.

There is overwhelming evidence that LDL-C is a causal contributor to the development of atherosclerosis (9). A previous study showed that LDL-C was a risk factor for the presence of any carotid plaque as well as for multiple plaques (16). Another study explored the correlation between blood lipid profile and atherosclerosis, finding that LDL-C was positively associated with the presence of carotid plaque (26). A large community-based cohort study suggested that elevated LDL-C was an independent predictor of carotid plaques among middle-aged German women (27). Similar findings were observed in this study. The results of this present study suggested that the level of LDL-C was an independent risk factor for increasing CIMT, even after adjustment for other risk factors. In other words, LDL-C was determined as a risk factor not only to the formation but also to the development of atherosclerosis. We also identified elevated LDL-C as a major determinant of atherosclerosis progress, and the multivariate analysis confirmed the increase in carotid plaque risk with increasing LDL-C levels in participants who were 45 years and older. This study corroborated previous results that found serum LDL-C to be an important risk factor for atherosclerosis, particularly in middle-aged individuals (28, 29). The underlying mechanism by which LDL-C leads to arterial atherosclerosis is well established. Once the endothelial cell layer is damaged, LDL-C is deposited on the arterial wall, leading to the formation of fatty streaks and foam cells, a hallmark of atherosclerotic lesions (30, 31). This also explained the results that LDL affected the entire process of atherosclerosis.

A high level of plasma HDL-C has long been believed to be associated with a reduced number of cardiovascular events in the general population as well as in those at high risk for cardiovascular events (32, 33). One study from Italy demonstrated that the HDL-C levels were inversely associated with the development/presence of atherosclerosis in humans (34). In addition, two studies have shown a significant inverse relationship between HDL-C and CIMT (35, 36). However, other studies found no significant relationship between HDL-C and CIMT (6, 37, 38), including one restricted to diabetic patients (6). In contrast to the aforementioned reports, our results suggested that high HDL-C was an independent protective factor against increased CIMT, but no significant association between the presence of carotid plaque and HDL-C was observed. Thus, HDL can delay the formation of atherosclerosis, but there was no effect to progress in atherosclerosis. Several possible mechanisms may explain this association between HDL-C and early-stage atherosclerosis. HDL-C is believed to inhibit the formation of atherosclerosis lesions in the arterial wall by removing excess cholesterol from cells and preventing cell adhesion and transmigration (39). These mechanisms may suggest that there is increased thickening of the arterial wall in individuals with low plasma HDL-C levels (40). However, the mechanisms underlying HDL-C's effect on the carotid plaque remain unclear.

While numerous previous studies have focused on CIMT, few have reported the effects of TC and TG on CIMT. Furthermore, the relationship between TG and CIMT remains controversial. The main finding of a study from Germany was that fasting TG was independently and positively associated with mean CIMT in acute ischemic stroke patients (41). Others have found no association between CIMT and fasting (42–44) or post-challenge TG (45). The present study demonstrated that CIMT increased with TC increase and TG decrease, while the risk of the carotid plaque increased with TC increase, but there were no significant differences between the carotid plaque and the LDL-C levels. In this study, although CIMT decreased with TG increase, there were no significant differences between TG and carotid plaque. Moreover, there was a thin CIMT in this low-income population, with a mean value of 567 μm. The precise relationship between CIMT and TG level needs to be explored by a long-term follow-up.

There are several limitations to this study. First, considering our study design, the inherent limitation of a cross-sectional study is that it cannot validate causal links between significant variables and CIMT; thus, further longitudinal studies are needed in order to determine causality. Second, the reliance on Chinese rural residents with low income and education levels may limit the generalizability of the study's findings. However, this rural population accounts for more than 50% of the total population of China; therefore, our results represent the findings in a significant population. In addition, we did not collect all information on medicine in all participants; however, due to their disadvantaged socioeconomic status, the frequency of medicine use was low and would not impact the validity of the results.

## Conclusions

In conclusion, the levels of TC, TG, HDL-C, and LDL-C associated significantly with the mean CIMT. High levels of TC and LDL-C and low levels of TG and HDL-C were the independent risk factors of early atherosclerosis. Nevertheless, increase in the levels of TC and LDL-C associated with a higher risk of late-stage atherosclerosis. These findings suggest that it is vital to manage and control the dyslipidemia standard levels in China, especially among rural residents, in order to reduce the burden of CVD.

## Data Availability Statement

The datasets analyzed in this article are not publicly available. Requests to access the datasets should be directed to Jinghua Wang, jhw8799@yahoo.com.

## Ethics Statement

The studies involving human participants were reviewed and approved by the ethics committee of Tianjin Medical University General Hospital; the methods were carried out in accordance with the approved guidelines, and informed consent was obtained from all participants. The patients/participants provided their written informed consent to participate in this study.

## Author Contributions

JW, QY, and XN contributed to the study design, performed the data interpretation, and critical review. JW performed the data analysis. JP, JL, and HW contributed to the drafting of the article. JP, JL, HW, WL, XD, QL, XZ, DQ, JT, XN, and JW performed the data collection, case diagnoses, and confirmation of case diagnoses. All the authors read, revised, and approved the final version of the paper.

## Conflict of Interest

The authors declare that the research was conducted in the absence of any commercial or financial relationships that could be construed as a potential conflict of interest.
